# Laboratory-developed Droplet Digital PCR Assay for Quantification of the JAK2 ^V617F^ Mutation

**DOI:** 10.1055/s-0044-1785537

**Published:** 2024-04-04

**Authors:** Yupeng Liu, Cong Han, Jie Li, Shicai Xu, Zhijian Xiao, Zhiyun Guo, Shuquan Rao, Yao Yao

**Affiliations:** 1 School of Life Sciences and Engineering, Southwest Jiaotong University, Chengdu, China; 2 State Key Laboratory of Experimental Hematology, National Clinical Research Center for Blood Diseases, Haihe Laboratory of Cell Ecosystem, Institute of Hematology & Blood Diseases Hospital, Chinese Academy of Medical Sciences & Peking Union Medical College, Tianjin, China; 3 Department of Hematopathololgy, Tianjin Institutes of Health Science, Tianjin, China; 4 Department of Laboratory Medicine, Peking University Shenzhen Hospital, Shenzhen, China

**Keywords:** myeloproliferative neoplasms, JAK2 V617F mutation, ddPCR, optimization

## Abstract

Precise quantification of the JAK2 ^V617F^ mutation using highly sensitive assays is crucial for diagnosis, treatment process monitoring, and prognostic prediction in myeloproliferative neoplasms' (MPNs) patients. Digital droplet polymerase chain reaction (ddPCR) enables precise quantification of low-level mutations amidst a high percentage of wild type alleles without the need for external calibrators or endogenous controls. The objective of this study was to optimize a ddPCR assay for detecting the JAK2 ^V617F^ mutation and establish it as a laboratory-developed ddPCR assay in our center. The optimization process involved fine-tuning five key parameters: primer/probe sequences and concentrations, annealing temperature, template amount, and PCR cycles. Our ddPCR assay demonstrated exceptional sensitivity, and the limit of quantification (LoQ) was 0.01% variant allele frequency with a coefficient of variation of approximately 76%. A comparative analysis with quantitative PCR on 39 samples showed excellent consistency (r = 0.988).

In summary, through rigorous optimization process and comprehensive analytic performance validation, we have established a highly sensitive and discriminative laboratory-developed ddPCR platform for JAK2 ^V617F^ detection. This optimized assay holds promise for early detection of minimal residual disease, personalized risk stratification, and potentially more effective treatment strategies in MPN patients and non-MPN populations.

## Introduction

The discovery of the JAK2 ^V617F^ somatic mutation has been a significant milestone in the molecular diagnosis of myeloproliferative neoplasms (MPNs), and the JAK2 ^V617F^ mutant alleles are detected in approximately 95% of patients with polycythemia vera (PV) and approximately 60% of patients diagnosed with essential thrombocythemia (ET) and primary myelofibrosis (PMF). 1 The JAK2 ^V617F^ mutant allele frequency is a key determinant of disease phenotype severity and progression in MPNs. 2 3 For instance, the JAK2 ^V617F^ mutant allele frequency is significantly associated with an elevated risk of thrombotic events in MPN patients, particularly among elderly individuals and those with higher hemoglobin levels in ET. 4 5 Moreover, both the JAK2 ^V617F^ mutation itself and its frequency holds significant prognostic value and survival implications, including their correlation with disease phenotype severity of PMF and progression to post-PV myelofibrosis. 6 7 In addition to MPN patients, detection, and quantification of the JAK2 ^V617F^ mutation in healthy individuals holds significant clinical significance as well. 8 9 The presence of JAK2 gene mutations in aging population was associated with increased risk of hematologic malignancies, all-cause mortality, and cardiovascular disease. 10 11 Moreover, healthy individuals with JAK2 ^V617F^ clonal hematopoiesis (CH) exhibited significantly elevated levels of white blood cell counts, platelet counts, and hemoglobin concentrations, as well as higher rates of arterial and venous thrombotic events compared to those without CH. 12 Additionally, the 2022 International Consensus Classification (ICC) of myeloid neoplasms and acute leukemias guideline strongly recommends sensitive detection of allele frequencies below 1%. 13 Thus, precise quantification of the JAK2 ^V617F^ mutant allele frequency is essential for disease diagnosis, progression monitoring, and prognosis assessment in both MPN and non-MPN populations.

Currently, despite the development of several molecular techniques, including real-time quantitative polymerase chain reaction (qPCR) which is widely utilized, there remains a lack of Food and Drug Administration (FDA)-approved tests available for the quantification of JAK2 ^V617F^ mutation. 14 15 16 However, it has been widely recognized that real-time qPCR results exhibit huge variability due to its susceptibility to sample quality and operator experiences and extensive efforts are required to enhance the standardization and assay quality. 17 Droplet digital PCR (ddPCR) is an innovative PCR-based technology that partitions the nucleic acid samples into thousands of nanoliter-sized droplets, and PCR amplification is carried out within each droplet, which enables absolute quantification of DNA copies. Sample partitioning mitigates the effects of target competition, making PCR amplification less sensitive to inhibition and greatly improving the discriminatory capacity of assays that differ by only single nucleotide. Additionally, the present-or-absent digital format and ability to draw clear thresholds between positive and negative droplet clusters at the end point of digital PCR means that we can absolutely quantify DNA without the need for external calibrations. In the current study, we aimed to establish a laboratory-developed ddPCR assay for accurate quantification of the JAK2 ^V617F^ mutation burden while ensuring high sensitivity and specificity.

## Materials and Methods

### Patients and Samples

A total number of 39 patients newly diagnosed or follow-up patients with MPNs including PV, ET, PMF, and 40 healthy donors with normal hemoglobin and platelet levels as negative controls were enrolled in this study. The samples (either bone marrow or peripheral blood) were collected between January 2021 and December 2023. Quantification of the JAK2 ^V617F^ mutation burden was performed on genomic DNA from bone marrow or peripheral blood samples. This study was approved by the Institutional Ethics Review Boards at the Institute of Hematology and Blood Diseases Hospital, Chinese Academy of Medical Sciences. Written informed consents were obtained from patients and healthy donors prior to sample collection.

The first World Health Organization (WHO) international reference panel for the JAK2 ^V617F^ mutation (The National Institute for Biological Standards and Control code: 16/120) was utilized as calibrators. This panel consists of seven different frozen genomic DNA samples with varying frequency of the JAK2 ^V617F^ mutation including 0, 0.0009, 1.00, 10.8, 29.6, 89.5, and 100%.

### Genomic DNA Preparation

Genomic DNA was isolated using the QIAamp DNA Mini Kit (QIAGEN) according to the manufacturer's protocol. The DNA concentration was determined using NanoDrop at a wavelength of 260 nm, and purity was estimated using the absorption ratio of DNA at a wavelength of 260 nm to that at a wavelength of 280 nm.

### Primer and Probe Design

The TaqMan MGB probes and primers were designed using Prime 5.0 software and synthesized by Sangon Biotech, Shanghai, China. The duplex assay consists of a forward primer, a reverse primer, and two probes—one for the JAK2 ^V617F^ mutant allele (c.1849G > T) and other for the wild type allele. The sequences of primers and probes used in this study are listed in Table 1 .

**Table 1:** Sequences of primers and probes

	Set name	Primer/Probe name	Sequence
ddPCR assay	set1	JAK2_V617F_WT_probe1	TCTCGTCTCCACAGACACATACT
		JAK2_V617F_Mut_probe1	TCGTCTCCACAGAaACATACTCC
		JAK2_F1	AGATGCTCTGAGAAAGGCATTAG
		JAK2_R1	TTGAAGCAGCAAGTATGATGAG
	set2	JAK2_F2	TGCTCTGAGAAAGGCATTAGAAAG
		JAK2_R2	GCAGCAAGTATGATGAGCAAGC
		JAK2_V617F_WT_probe2	CCACAGACACATACT
		JAK2_V617F_Mut_probe2	TCCACAGAAACATAC
allele-specific qPCR assay	set1	Common forward primer	CTTTCTTTGAAGCAGCAAGTATGA
		Common probe	TGAGCAAGCTTTCTCACAAGCATTTGGTTT
		Wild type-specific reverse primer	GTAGTTTTACTTACTCTCGTCTCCACATAC
		JAK2V617F mutation-specific primer	GTAGTTTTACTTACTCTCGTCTCCACATAA

Abbreviations: ddPCR, digital droplet polymerase chain reaction; qPCR, quantitative polymerase chain reaction.

### Droplet Digital Polymerase Chain Reaction

Optimized reactions were performed in 20 μL of duplex ddPCR reaction mix containing 2× ddPCR Supermix for Probes (Bio-Rad, United States), forward and reverse primers at a final concentration of 450 nM, wild type and mutant probes at a final concentration of 250 nM, as well as template genomic DNA. The PCR was performed in a Thermocycler T100 (Bio-Rad, United States) with the following protocol: initial denaturation at 95°C for 10 minutes (1 cycle), followed by denaturation at 95°C for 15 seconds and annealing at 57°C for 60 seconds (40 cycles), incubation at 98°C for additional 10 minutes (1 cycle), and final cooling to 12°C.

The ddPCR assay was performed using the QX200 AutoDG Droplet Digital PCR System (Bio-Rad, United States) according to the manufacturer's instructions. Positive and negative droplets were counted using the QX200 Droplet Reader (Bio-Rad) and analyzed by Poisson statistics using the QX Manager Software (standard edition, Bio-Rad, United States).

### Limit of Blank

The limit of blank (LoB) assessment was conducted in accordance with the experimental plan recommended by Clinical and Laboratory Standards Institute (CLSI) EP17-A2. Specifically, water was used as no-template control (NTC), and genomic DNA (100 ng each) from healthy individuals was used as negative control. A total of 40 NTC and 40 negative controls were prepared. The ddPCR of negative controls was repeated twice on one ddPCR system by a single operator, utilizing two independent lots of synthesized primer/probe reagents. This resulted in a total of 80 reactions for the negative samples and 40 reactions for the NTC. LoB was calculated as follows.



where *c _p_* is a multiplier to obtain the 95th percentile of a normal distribution, *B* represents the total number of blank results in the dataset, and *K* denotes the number of blank and negative samples.

### Analytical Precision and Accuracy

Within run (intra-assay), between run (interassay), between day (interassay), and total precision were assessed by testing the JAK2 ^V617F^ -positive patient samples with varying frequencies which had been previously quantified by real-time qPCR. Each sample was tested by two operators on a single instrument. All samples were tested in triplicate. The mean, standard deviation (SD), and coefficient of variation (CV) were calculated for each sample according to the CLSI EP5-A3-prescribed methods for data analysis.

To assess the accuracy of copy numbers, a set of seven genomic DNA standards with the JAK2 ^V617F^ mutation at varying frequency (0, 0.0009, 1.00, 10.8, 29.6, 89.5, and 100%) from WHO international reference material (No. 16/120) was tested by ddPCR. Each DNA standard was tested in 10 replicates by a single operator on three consecutive days.

### Analytical Sensitivity and Limit of Detection

We followed the CLSI EP17-A2 guidelines (Evaluation of Detection Capability for Clinical Laboratory Measurement Procedures by Classic approach) to assess limit of detection (LoD), with a type 2 error set at 5%. LoD is defined as the lowest percentage of the JAK2 ^V617F^ mutation that can be reliably detected. Specifically, genomic DNA from wild type and patient samples were mixed to prepare test samples with the frequency of the JAK2 ^V617F^ mutation at approximately 0.01 and 0.005%. Samples were tested in 20 replicates by one operator on one instrument for three consecutive days, resulting in a total of 240 tests.

### Linearity

The assay linearity was determined by measuring dilution series prepared using WHO international reference materials with wild type negative samples. A series of variant allele frequency (VAF) values of 100, 10.8, 1, 0.03, 0.01, and 0.005%, were generated and tested in four replicates. Weighted least squares regression analysis (WLS) was conducted in accordance with the CLSI EP06 2nd edition requirements for evaluating linear range performance of quantitative experiments.

### Allele-specific Quantitative Polymerase Chain Reaction

The allele-specific qPCR assay was performed as described previously. 15 The assay utilized a common forward primer and a common probe along with a wild type-specific reverse primer and the JAK2 ^V617F^ mutation-specific primer. Each qPCR consisted of 50 ng template DNA in a total volume of 25 μL. The reaction mixture included 12.5 μL TaqMan Universal PCR Master Mix (with UNG and ROX) and 2.5 μL of 10× primer/probe mixture, with final concentrations of 450 nM for the primers and 250 nM for the probe. The PCR program included an initial step at 50°C for 2 minutes, followed by denaturation at 95°C for 10 minutes, then proceeded with 40 cycles of denaturation at 95°C for 15 seconds and annealing/extension at 60°C for 60 seconds. The Applied Biosystems 7500 Sequence Detection System was used for the PCR, and data analysis was performed using the 7500 System version 1.4.0 software (Applied Biosystems, United States).

### Statistical Analysis

Statistical analysis and plotting were performed using the R programming language where *p* < 0.05 indicated difference of statistical significance between groups. Consistency analysis was conducted using the Bland–Altman method. Pearson correlation analysis was used to calculate the correlation coefficient (r). Linear regression analysis was employed to assess the accuracy in measurement results compared with the certificated value defined by WHO reference material.

## Results

### Optimization of Digital Droplet Polymerase Chain Reaction Experimental System

**Fig. 1:**
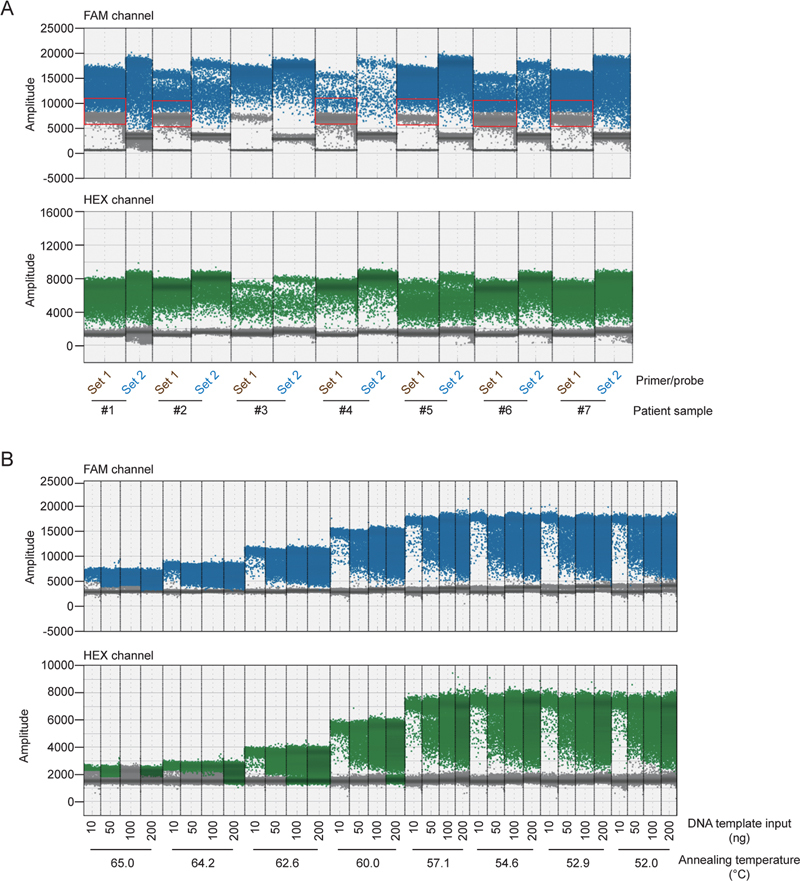
Optimization of JAK2 ^V617F^ ddPCR assay for two sets of primer/probe and annealing temperature. ( **A** ) Patient samples from seven different VAFs (#1–#7) were examined using two sets of primers/probes (set1 and set2). The FAM channel showed JAK2 ^V617F^ positive (green) and negative (black) droplets, and the HEX channel showed JAK2 wild type positive (green) and negative (black) droplets. Negative and positive droplet separation was good in the HEX channel, but JAK2 ^V617F^ positive and negative droplet in the corresponding FAM channel had poor separation effect when set2 primers/probes were used (red box). ( **B** ) Using different DNA template inputs (10, 50, 100, 200 ng) in the annealing temperature gradient, the negative and positive droplet separation in the two channels changed as the conditions changed. When the annealing temperature was 57.1°C and the template DNA input was 10 ng, the negative and positive droplets in the two channels were best separated. ddPCR, digital droplet polymerase chain reaction; VAF, variant allele frequency.

We designed two sets of primer/probe (set1 and set2) for ddPCR to detect the JAK2 ^V617F^ mutant allele and the wild type JAK2 allele ( Table 1 ). It was observed that set2 primers/probes demonstrated superior separation between the positive and negative droplets at both FAM fluorescence channel (JAK2 ^V617F^ mutant allele) and HEX fluorescence channel (wild type JAK2 allele; Fig. 1A ). The ddPCR experimental parameters were then optimized, with a particular focus on the annealing temperature, in order to enhance the analytical performance specifically for set2 primers/probes. The difference of mean amplitude (fluorescence signal) between the positive and negative droplets (hereafter referred to as “mean amplitude difference”) was applied to quantify the ddPCR performance. The optimal annealing temperature for the JAK2 ^V617F^ duplex ddPCR was determined through gradient PCR across an annealing/extension temperature ranging from 52 to 65°C. Remarkably, a temperature of 57.1°C consistently yielded robust fluorescence signals from positive droplets with maximum mean amplitude difference ( Fig. 1B ). Therefore, an annealing temperature of 57.1°C was selected in the subsequent experiments.

**Fig. 2:**
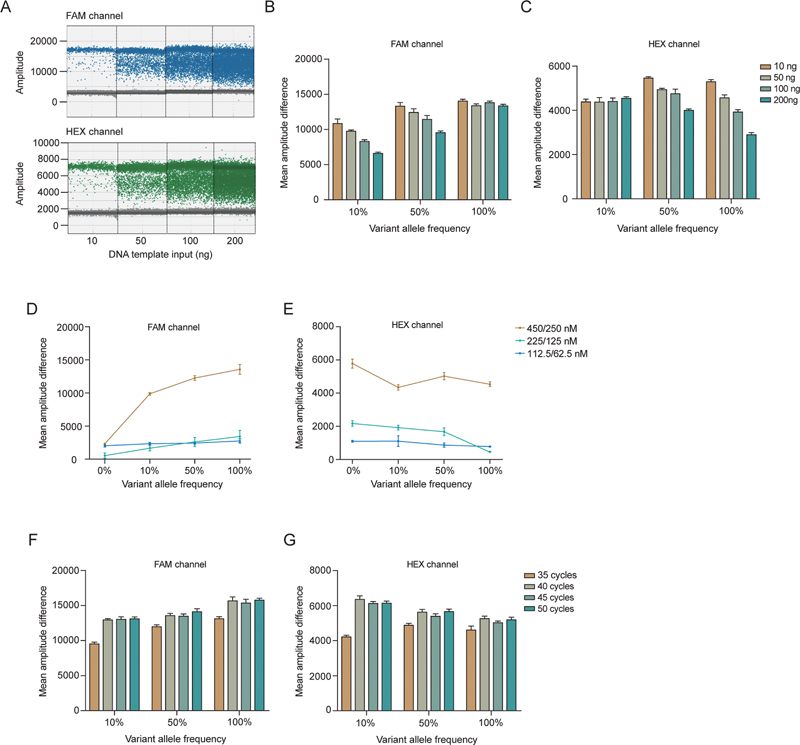
Optimization results of template DNA input, primer/probe concentration, and cycle number of LDT **-** ddPCR system. ( **A** ) The ddPCR system is a direct result of the separation of negative and positive droplet in two channels at different template dosage (10, 50, 100, 200 ng). It can be seen that the negative and positive droplet separation effect of the two channels is the best when the template input is about 10 ng. ( **B** , **C** ) Templates with different VAFs (10, 50, 100%) were used to detect the separation of negative and positive droplets in the two channels at different doses (10, 50, 100, 200 ng), and the histogram was quantified by the mean amplitude difference. ( **D** , **E** ) Different VAF templates (0, 10, 50, 100%) were used to detect the separation of negative and positive droplets in the two channels at different primer/probe concentrations (450/250, 225/125, 112.5/62.5 nM), and the line plots were quantified by the mean amplitude difference. ( **F** , **G** ) Different VAF templates (10, 50, 100%) were used to detect the separation of negative and positive droplets in the two channels at different cycles (35, 40, 45, 50 cycles), and the histogram was quantified by the mean amplitude difference. ddPCR, digital droplet polymerase chain reaction; LDT, laboratory-developed test; VAF, variant allele frequency.

Next, we investigated the performance of ddPCR using varying amounts of DNA template. As the amount of DNA template increased, there was a corresponding increase in the number of droplets detected on both the FAM and HEX channels ( Fig. 2A–C ). While larger amount of DNA template could enhance the probability to detect the JAK2 ^V617F^ mutant allele of low frequency, surpassing a certain threshold posed challenges in terms of dynamic range limitations and distinguishing between positive and negative droplets ( Fig. 2A–C ). To achieve an optimal balance between sensitivity and signal intensity, a 100-ng genomicDNA template input was employed in the subsequent assays, unless otherwise specified.

Additionally, we have also explored the effect of primer/probe concentrations and PCR cycles on analytic performance. We performed ddPCR using different final concentrations of primers (450, 225, and 112.5 nM), with the probe concentration half that of the primers. The highest mean amplitude difference was achieved when using primer/probe at a concentration of 450/225 nM regardless of the VAF ( Fig. 2D, E ). Finally, we explored the optimized PCR cycles and found that increasing PCR cycles from 35 to 40 increased the mean amplitude difference ( Fig. 2F, G ). However, further increase of PCR cycles did not result in higher mean amplitude difference ( Fig. 2F, G ). Therefore, we utilized primer/probe concentrations at 450/225 nM and conducted a total of 40 cycles in subsequent ddPCR assays.

In sum, the optimized ddPCR condition was as follows: annealing temperature at 57°C, DNA template amount of 100 ng, primer/probe concentrations of 450/250 nM, and a total of 40 PCR cycles.

### Precision and Accuracy

Following the CLSI EP05-A3 guidelines, the laboratory-developed ddPCR method was utilized to analyze DNA samples harboring varying frequencies of the JAK2 ^V617F^ mutant allele. As demonstrated in Table 2 , the ddPCR method accurately detected the JAK2 ^V617F^ mutant allele at different frequencies in a wide range (0.1, 1, 10, and 100%), exhibiting a CV ranging from 0.25 to 20.17% (total precision in Table 2 ). Moreover, the ddPCR results demonstrated excellent repeatability, with SD ranging from 0.03 to 0.36% (total precision in Table 2 ). The interoperator and interday reproducibility yielded similar outcomes as well ( Table 2 ).

**Table 2:** Precision

Target VAF	Intra-assay (within run) precision			Interoperator precision			Interassay (between day) precision			Total precision		
	Mean VAF (%)	SD (%)	CV (%)	Mean VAF (%)	SD (%)	CV (%)	Mean VAF (%)	SD (%)	CV (%)	Mean VAF (%)	SD (%)	CV (%)
100%	93.828	0.074	0.079	93.813	0.262	0.28	93.752	0.241	0.257	93.775	0.234	0.25
10%	14.327	0.221	1.545	14.154	0.297	2.1	14.137	0.337	2.381	14.127	0.362	2.565
1%	1.306	0.048	3.676	1.368	0.069	5.046	1.346	0.05	3.687	1.325	0.052	3.911
0.10%	0.163	0.011	6.5	0.15	0.036	23.916	0.144	0.024	16.79	0.146	0.029	20.174

Abbreviations: CV, coefficient of variation; SD, standard deviation; VAF, variant allele frequency.

**Fig. 3:**
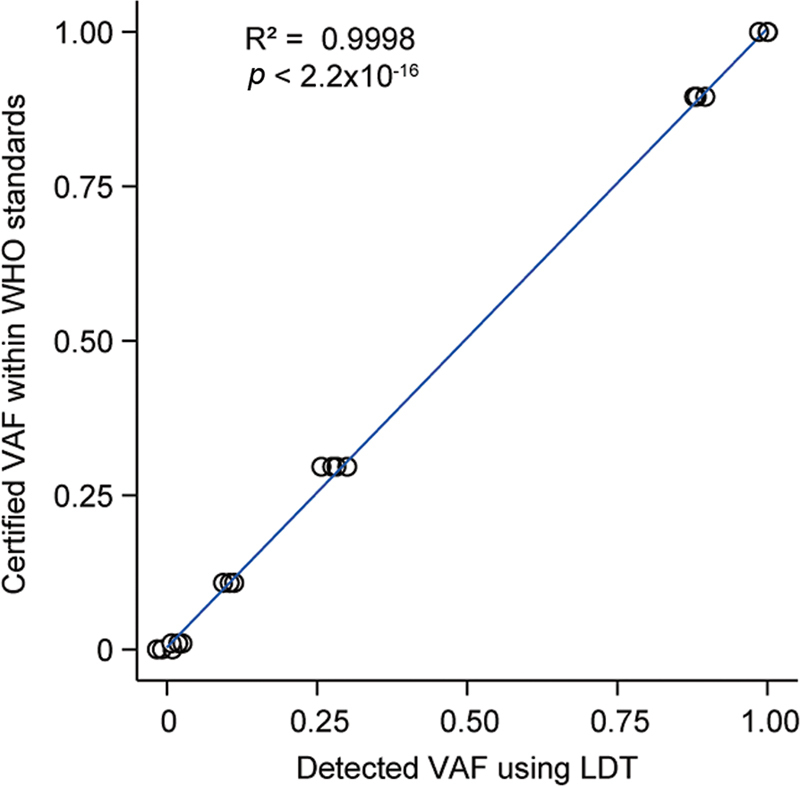
Accuracy evaluation of ddPCR assay on the WHO reference products. The WHO international reference product (No. 16/120) provide value (0, 0.0009, 1.00, 10.8, 29.6, 89.5, 100%) was correlated with the mean value of WHO international reference for detecting JAK2 ^V617F^ mutation by LDT-ddPCR, *R* ^2^ = 0.9998 ( *p* < 2.2 × 10 ^−16^ ). No less than 10 repetitions for each level, a total of 70 tests. ddPCR, digital droplet polymerase chain reaction; VAF, variant allele frequency.

We further evaluated the accuracy of our laboratory-developed ddPCR method using WHO international reference material (No. 16/120), which encompasses various frequencies of the JAK2 ^V617F^ mutant allele. Briefly, our ddPCR method accurately detects the frequency of JAK2 ^V617F^ mutant allele within the international reference material (No. 16/120), and regression analysis demonstrated a y-intercept of −0.210141 with a slope equal to 0.9987, resulting in an *R* ^2^ value of 0.9998 ( Fig. 3 ). The variation across the replicates, as reflected by CV percent, ranged from a maximum of 20.186% at an extremely low level such as 0.031% VAF to less than 1% at higher VAF values exceeding 1%.

### Analytical Specificity and Limit of Blank

The LoB was calculated utilizing parametric statistical methods to establish the minimal detectable amount for JAK2 ^V617F^ mutant allele presence. The JAK2 ^V617F^ mutant quantification ddPCR assay we established exhibits a LoB value of 0.002% VAF, 0.043 copies/μL, with a minimum requirement of more than one positive droplet.

### Analytic Sensitivity and Limit of Detection

According to the recommendations of CLSI EP17-A2, we prepared DNA samples with extremely low frequencies of the JAK2 ^V617F^ mutant allele, 0.01 and 0.005%. We then utilized our ddPCR method to analyze these DNA samples in a 20-μL PCR system with 100-ng DNA input. Each assay was repeated 20 times. Our analysis resulted in an LoD of 0.005% VAF with a CV of approximately 76%. The concentration-based LoQ was determined as well, which was demonstrated to be as low as 0.01% VAF, with a concentration of 0.193 copies/μL in a 20-μL system.

### Linearity

**Fig. 4:**
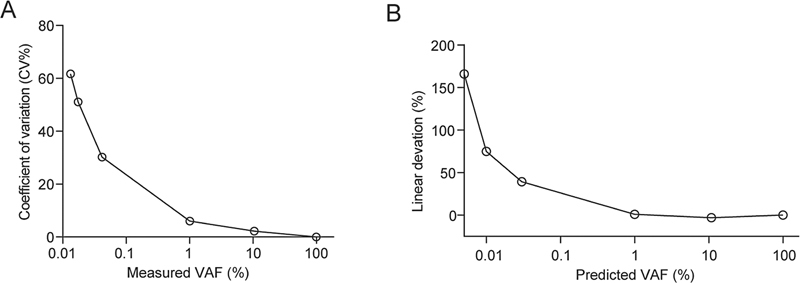
The linear range of JAK2 ^V617F^ allele mutation frequency (VAF) of ddPCR assay. ( **A** ) Scatter map of CV values of VAF measurements. ( **B** ) Scatterplot of the percentage deviation from the measured value and predicted value of LDT-ddPCR. CV, coefficient of variation; ddPCR, digital droplet polymerase chain reaction; VAF, variant allele frequency.

According to the WHO international reference material (No. 16/120), we mixed wild type DNA with that of the JAK2 ^V617F^ mutation to obtain DNA samples with VAF levels of 100, 10.8, 1, 0.03, 0.01, and 0.005%. Following the requirements outlined in CLSI document EP06 (2nd edition) for evaluating linear range performance in quantitative experiments, we subjected the ddPCR results to WLS. The criterion used for determining linearity was that the CV values for VAF should be less than 76% and the allowable absolute linear deviation percentage should be less than 100%. 18 19 Our laboratory-developed ddPCR method demonstrated a linear range from as low as 0.01% up to l00.0% for detecting the frequency of JAK2 ^V617F^ mutant allele, with a maximum CV and linear deviation percentage of 51.09 and 74.93%, respectively ( Fig. 4 ). Moreover, using our ddPCR method consisting of 50 ng DNA template in a 20-μL reaction system, we demonstrated a copy number linear range between 3.86 and 23246.42.

### Comparison between Digital Droplet Polymerase Chain Reaction and Real-time Quantitative Polymerase Chain Reaction

**Fig. 5:**
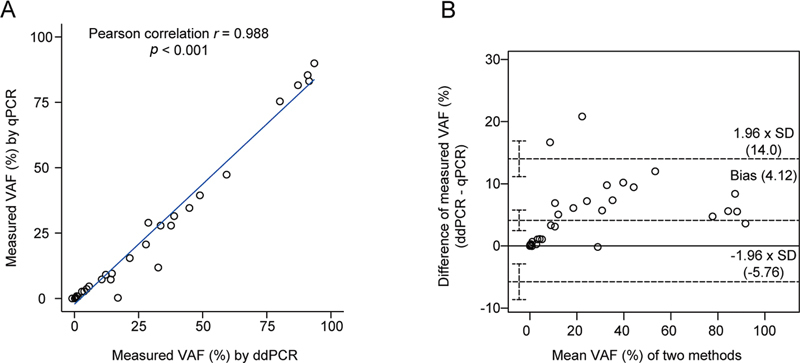
Comparison of ddPCR and qPCR. ( **A** ) JAK2 ^V617F^ of 39 samples was detected by LDT-ddPCR and qPCR, respectively. Pearson correlation analysis of the detected results showed that the Pearson correlation coefficient *r* = 0.988, *p* < 0.001. ( **B** ) Bland–Altman scatter plot, the middle dotted line is the mean difference between the mean value of JAK2 ^V617F^ detected by LDT-ddPCR method and the mean value detected by qPCR, and the upper and lower two dotted lines represent the 95% upper and lower limits of the consistency limit. CV, coefficient of variation; ddPCR, digital droplet polymerase chain reaction; qPCR, quantitative polymerase chain reaction; VAF, variant allele frequency.

A total of 39 DNA samples from patients with varying degrees of the JAK2 ^V617F^ mutation allele frequency were collected. Overall, we observed a strong correlation between the JAK2 ^V617F^ mutant allele frequency obtained from both the ddPCR and real-time qPCR method (Pearson's correlation r = 0.988, *p* < 1 × 10 ^−4^ ; Fig. 5A ). Our ddPCR method accurately detected the frequencies of JAK2 ^V617F^ mutant allele in all 39 patients, with a minimal detectable VAF of 0.018%. In contrast, the real-time qPCR assays fail to detect frequencies below 0.05%, resulting in four patients that can only be detected using the ddPCR method. Furthermore, we employed Bland–Altman analysis to assess any systematic differences in variance between the two methods. For the evaluated paired samples ( *N* = 39), Bland–Altman analysis revealed a mean bias of 4.12% for ddPCR/qPCR comparison ( *p* < 1 × 10 ^−4^ ; Fig. 5B ). The limits of agreement (−5.76 to 14%) represent the interval that is expected contain 95% of data points would fall based on an assumed normal distribution, as depicted by dashed grey lines. Lastly, it is worth noting that there were comparable medians for the JAK2 ^V617F^ mutant allele quantification from both the ddPCR and real-time qPCR methodologies.

## Discussion

The JAK2 ^V617F^ mutation plays a pivotal role in MPNs, affecting clinical presentation, prognosis, and response to targeted therapies. Precise quantification of its allelic burden is crucial for optimal patient management. However, existing detection methods often lack sensitivity and/or reproducibility, particularly when dealing with minimal residual disease or low mutation frequencies. In this study, we addressed this challenge by establishing a highly sensitive and discriminative laboratory-developed ddPCR assay for JAK2 ^V617F^ quantification. This enhanced sensitivity holds promise for earlier detection of mutants in MPN patients and the measurement of the low JAK2 ^V617F^ allele burden could offer new prognostic parameters.

Digital PCR was developed to provide high precision, absolute quantification of nucleic acid targets for both research and clinical diagnostic applications. Given the exceptional precision achievable with ddPCR, it is crucial to incorporate a comprehensive experimental design that encompasses all known sources of variation and includes bias components in the uncertainty budget to ensure accurate measurements. 20

The primer and probe sets for JAK2 ^V617F^ ddPCR were initially compared in this study, and set2 was subsequently chosen for further optimization. To achieve superior assay performance, we meticulously optimized five key parameters of ddPCR. Specifically, we carefully selected five parameters that are most important for ddPCR detection, including primer/probe combinations, annealing temperature, template input amount, primer/probe concentration, and number of cycles, and then tested the reaction system under various conditions. An annealing temperature of 57°C provided optimal discrimination due to its excellent separation between positive and negative droplets and a high signal-to-noise ratio at both detection channels.

Additionally, two key factors influence the reliability of digital PCR measurements: the number of reactions or partitions analyzed and the number of template molecules in the assay. 21 QX200 ddPCR system can accommodate up to 120,000 copies in a 20-μL reaction, which is equivalent to the amount of 400 ng human genomic DNA, assuming one copy per haploid genome. The binomial approximation and system error are taken into consideration, leading Bio-Rad to recommend an upper concentration limit of five copies per droplet on the QX200 ddPCR platform. This recommendation ensures a theoretical dynamic range of 10 ^5^ target copies. In our experiments, we observed up to 5% of empty droplets at high concentrations of DNA samples (200 ng DNA in a 20-μL reaction), which indicates that the practical average copies per droplet would exceed theoretical calculations. We propose that a template input amount of 100 to 200 ng demonstrates an optimal balance between dynamic range and sensitivity. Furthermore, this range ensures high precision as it aligns with the peak precision observed in a previous study where there were approximately 1.59 copies per droplet. 22 23 The recommended amount of DNA template for newly diagnosed patients is 50 ng, and for follow-up patients it is 100 ng, considering the need to balance detection capability for rare variants and the dynamic range limitations of the QX200 ddPCR system. Moreover, effective use of dilutions enables an increase in the overall dynamic range as seen in a previous study. 24 The signal value and separation are significantly enhanced with a primer/probe concentration of 450/250 nM, compared to concentrations of 225/125 and 112.5/62.5 nM, striking the ideal balance between specificity and signal strength. Moreover, increasing the cycle number to 40 amplifies the signal value and separation even further compared to 35 cycles, demonstrating its suitability for detecting low frequency and rare mutant alleles in the samples within the droplets of the reaction system.

Starting from the experiment conditions determined above, a comprehensive analytic performance of JAK2 ^V617F^ mutation ddPCR quantification assay was performed in our study. The ddPCR assay demonstrated a high level of precision, with a CV as low as 0.25% for higher VAF (approximately 100%) and 20.17% for a lower VAF value of 0.1%. According to CLSI experiment guidelines, LoB and LoD values were determined by parametric statistical methods. LoB value was 0.002% VAF and 0.043 copies/μL, with a minimum requirement of more than one positive droplet. LoD was 0.005% VAF with a CV of approximately 76%, while LoQ was set at 0.01% VAF, corresponding to a concentration of 0.193 copies/μL in a 20-μL system. The LoD reported in the previous studies 25 26 27 28 29 ranged from 0.007 to 0.01%. In comparison, our laboratory-developed ddPCR assay exhibited a slightly enhanced LoD for quantifying JAK2 ^V617F^ mutation, as compared to previous reports. The linear range for detecting the frequency of JAK2 ^V617F^ mutant allele spanned from 0.01 to l00.0%, with a maximum CV and linear deviation percentage up to 51.09 and 74.93%, respectively. Setting a criterion for a required level of precision effectively imposes a constraint on the dynamic range but ensures a minimum level of precision across that range. 30

Several molecular methods have been proposed for detecting the JAK2 ^V617F^ mutation, including Sanger sequencing, pyrosequencing, and high-resolution melting curve analysis, among others have shown limited sensitivity of typically less than 5% and some of these methods are not quantitative. Allele-specific qPCR can achieve sensitivities lower than 1% (1% cutoff in our center) and is therefore widely used for JAK2 ^V617F^ mutation quantification. A high consistency between the ddPCR and qPCR was shown in our study, which is similar to previous studies. 27 The JAK2 ^V617F^ mutation was detected at a higher frequency in clinical samples using ddPCR compared to qPCR, suggesting the potential of our optimized assay for early detection of minimal residual disease, personalized risk stratification, potentially more effective treatment strategies and relapse in MPN patients. Furthermore, while both qPCR and ddPCR demonstrated high sensitivity, enabling to detect the mutant allele burden at diagnosis, also below 1%, the ddPCR demonstrated higher precision at lower level mutation burdens.

In addition to the utility of diagnosis and prognosis for MPN patients, our platform can contribute to precise quantification of JAK2 ^V617F^ somatic mutation of CH of indeterminate potential in the general population, which typically exhibits a low mutation allele burden. 26 The recent study from Danish General Suburban population screening research, found that a JAK2 ^V617F^ population prevalence of 3.1% with a mean allele burden of 2.1%. Additionally, the majority of the population has an allele burden less than 1%. 26 The assessment of low JAK2 ^V617F^ mutation allele burdens in healthy individuals in these cases could serve as a crucial indicator for early detection of clinical progression to disease in at-risk individuals. Furthermore, ddPCR could detect low allele burdens as a sign of the presence of a small, mutated clone within an overall polyclonal hematopoiesis, which may arise independently and finally lead to myeloproliferative disease. 31 32

In summary, our optimized assay, with an annealing temperature of 57°C, template input of 50 to 100 ng, and primer/probe concentration of 450/250 nM, achieves the LoD of 0.005%, the LoQ of 0.01% mutant allele burden, significantly surpassing the sensitivity of conventional methods. This optimized assay facilitates more precise diagnosis, personalized treatment, and potential advancements in therapeutic development for MPN and non-MPN population. Future studies could explore the utility of ddPCR in assessing early preclinical stages of MPN development and investigate the correlation between JAK2 ^V617F^ allelic burden and specific clinical phenotypes.
